# Oral Health Knowledge, Attitude, and Practices among School Teachers in Chitwan District, Nepal

**DOI:** 10.1155/2021/9961308

**Published:** 2021-10-04

**Authors:** Harender Singh, Sanjeeb Chaudhary, Abhishek Gupta, Anusha Bhatta

**Affiliations:** ^1^Department of Public Health Dentistry, Chitwan Medical College, Bharatpur, Chitwan 44207, Nepal; ^2^Department of Conservative Dentistry and Endodontics, Chitwan Medical College, Bharatpur, Chitwan 44207, Nepal; ^3^Department of Oral Medicine and Radiology, Chitwan Medical College, Bharatpur, Chitwan 44207, Nepal; ^4^Kathmandu Medical College, Kathmandu 44600, Nepal

## Abstract

**Background:**

Oral health is dependent on the knowledge and awareness of the individuals. Teachers as individuals influence the children in every aspect including oral health maintenance. The objective of this study was to assess the oral health knowledge, attitude, practices, and oral health status among school teachers in Chitwan District, Nepal.

**Materials and Methods:**

A cross-sectional study was conducted on 550 school teachers of private and government schools in Chitwan District, to assess the knowledge, attitude, practices, and oral health status. Descriptive analysis was done and data were analyzed using chi-square. A five-point Likert Scale was applied to compute knowledge, attitude, and practices of the school teachers.

**Results:**

Most of the school teachers had good knowledge about periodontal diseases in comparison to dental caries. Private school (20.7% : 57) teachers had higher good oral health knowledge as compared to government school teachers (9.8% : 27). An almost equal percentage of private and government teachers (73.5% : 202 and 74.2% : 204, respectively) were found with fair knowledge about oral health. Males had a higher percentage for good oral health knowledge as compared to females.

**Conclusion:**

There is an incredible need to improve oral health knowledge and attitude among school teachers concerning different problems of the oral cavity and the avoidance of dental diseases. These enhancements can be applied through regular training of teachers by oral health seminars, materials, and other such types of methods.

## 1. Introduction

Oral health is a primary division of overall health. As the oral cavity is the doorway for the human being body, any damage to oral health can be evident not only in the oral cavity but also in other parts of the body. As prevention is better than cure, preventive procedures are being executed within various divisions of society [1]. According to a report of the World Oral health Organization (WHO), Global Data Bank (1995), over 15% of the nations in the globe confirm an average of 4.5 decayed, missing, or filled teeth for each child up to 12 years old. To manage the growing load of oral diseases, some developing countries freshly introduced school-based oral health education and prevention programs which intend to improve oral health behavior and status of the child population [2]. In Nepal, the 2004 National Pathfinder Survey shows that 58% of 5-6-year-old school children suffer from dental caries. With the caries prevalence of 58%, dental caries is more prevalent than malnutrition which affects 49% of the child population [3]. The benefits of using teachers include the capacity to reach all children, increased stability, improved communication, lower activity costs, and, most importantly, the fact that the teacher is one of the primary leaders during the early stages of personality development [4].

Since not many studies have been stated in the literature particularly in a third world country, an effort is made to assess the oral health practices among private and government school teachers. The aim of this study was to evaluate the oral health knowledge, attitude, and practices of government and private school teachers. This study would offer basic information for the planning and evaluation of school-based oral health promotion programs.

## 2. Materials and Methods

A cross-sectional survey was conducted among 550 govt. and private school teachers of Chitwan district, selected by stratified random sampling method. The study took place over four months from 16th October 2020 to 15th January 2021. Ethical clearance was obtained from the Institutional Review Committee (CMC-IRC/077/078-055) before starting the study. A total of 4 subject experts and 4 junior dentists were involved in conducting the study. Data were collected by four trained junior dentists under the supervision of a principal investigator. Three subject experts were involved in the preparation of the questionnaire and also to pretest its reliability. A self-administrated closed-ended questionnaire had been given to fill in the required information. The questionnaire had two components, general information as regards demography of the study population, for example, name, age, sex, address of the school where they were working, type of the school, teaching experience and educational qualification, and questions related to knowledge, attitude, and practices. A five-point Likert Scale was applied to compute knowledge, attitude, and practices of the school teachers. A pilot study was conducted on 50 subjects, selecting by applying simple random sampling, to check for the validity of the questionnaire and operational feasibility. Cronbach's alpha was applied for the reliability. Data were analyzed using SPSS software version 23. Data analysis began with tabulation of results. The values were represented in number, %, and mean ± SD. The *χ*^2^ test was used to test the significant differences in proportions. A written consent was acquired from the school authorities and all the school teachers before the commencement of the study.

### 2.1. Inclusion Criteria

All the teachers working in the selected school and agreeing to participate in the study were included in the study.

### 2.2. Exclusion Criteria

Teachers, not present on the scheduled date of the survey or not agreeing to participate, were excluded from the study.

## 3. Results

The response rate of the present study is 100 percent because all the school teachers agreed on the study and there was no mistake in filling in the questionnaire, so nothing was rejected.

Table 1 represents the distribution of the study population according to age and gender. The age of the teachers ranged from 21 to 50 years with a mean age of 35.53 ± 11.5 years. A total of 550 teachers were included for the study.  Table 2 indicates the genderwise distribution of the study population according to types of school.

Table 3 gives the distribution of school teachers based on knowledge and type of school. Private school (20.7% : 57) teachers had higher good oral health knowledge as compared to government school teachers (9.8% : 27). Almost equal percentages of private and government teachers (73.5% : 202 and 74.2% : 204, respectively) were found with fair knowledge about oral health.

Table 4 gives the distribution of school teachers based on knowledge and gender. The majority of males (68.5%) and females (77.2%) had fair oral health knowledge. Males had a higher percentage for good oral health knowledge as compared to females. Similarly, females had a higher percentage than males regarding poor knowledge about oral health. A significant difference was found regarding knowledge about oral health based on gender.

Table 5 depicts the distribution of school teachers based on their practices and type of school. The government school teachers had higher (19.3% : 53) good oral health practices as compared to private school teachers (4% : 11). On the other hand, private school teachers had higher fair and poor oral health practices as compared to government school teachers. A significant difference was found between private and government teachers on the basis of practices and type of school.

Table 6 gives the distribution of school teachers based on their attitude and type of school. Among private school teachers, the majority (72.7% : 200) had a fair oral health attitude. Similarly, among rural school teachers, the majority (78.2% : 215) had a fair oral health attitude. A little percentage of private and government teachers (13.5 : 37% and 9.5% : 26, respectively) had poor oral health. 13.8% (38) private and 12.4% (34) government teachers had good oral health. However, the percentage was found almost similar for the oral health of private and government teachers; thus, the difference was nonsignificant (*P*value <0.05) as shown in Table 7.

## 4. Discussion

A total of 550 participants were in the study, including 38.7% (213) of males and 61.3% (337) of females. Among private school teachers, the majority were females (207, 75.3%) and 24.7% (68) were males. Among government school teachers, 52.7% (145) were male teachers and 47.3% (130) were females. The observations are similar to the study done by Manjunath and Kumar [5] (2013), in which there was a higher percentage of female teachers than male teachers. The reason could be because, in a country like India, there are disparities in education between private and government schools.

The age of the teachers ranged from 21 to 50 years with a mean age of 35.53 ± 11.5. Based on the educational status of the school teachers, they were grouped as undergraduate 8.54% (47), graduate 47.63% (262), and postgraduate 43.81% (241).

54% of school teachers think gum bleeding means inflamed gums that is much similar to the previous study done by Manjunath and Kumar [5] in 2013. It was proposed that school teachers have a fair understanding of gum bleeding. According to another study conducted by Lang and Wolfolk [6] in 2001, 43.82% of school instructors feel that vitamin C protects gingival tissues from bleeding. The reason for this could be that vitamin C or citrus fruits are thought to help prevent gum bleeding in the past, and most school teachers believe that vitamin C is more effective than other materials.

However, only 40.54% said that using a toothbrush, paste, and floss protects gums from bleeding, whereas in another study by Lang and Wolfolk [6], 69.6% said that brushing and flossing prevent gum diseases, which is higher than the current study, which shows that school teachers' oral health knowledge is lacking. Plaque, according to 31.6% of school teachers, refers to soft material on teeth, which is similar to another study conducted by Manjunath and Kumar [5].

When asked what dental plaque causes, 40% of school teachers said tooth discoloration, followed by 20% who said dental caries, which is low compared to Elena and Petr's study [7], which could be related to a lack of understanding about dental plaque.

47.8% of school teachers think dental decay means a hole appears in teeth and 37.8% of teachers responded with a black spot on the tooth surface that is similar to the study done by Manunath and Kumar [5]. We can say school teachers have average knowledge about dental decay.

According to Al-Tamimi and Petersen [8], about 78.2% of respondents believe that sugar consumption has an impact on oral health. The reason for this in the current study is a lack of awareness about dental plaque, and many teachers still believe sugar causes dental problems as compared to other elements of the oral cavity, which is comparable to the general public in India. Another explanation for this could be because many newspapers and television stations now report that sugar consumption is hazardous to a person's oral cavity as well as overall health.

76% feel that oral and dental health improves overall health, which is similar to another study conducted by Zhu et al. [9] According to other surveys, 86.6% of people feel that cleaning their teeth properly reduces dental decay.

Fluoride makes teeth stronger and reduces dental decay, according to 47.2% of respondents, which is significantly lower than other studies conducted elsewhere by Elena and Petr [7]. The explanation for this could be a lack of understanding and encouragement by dentists in our country about the beneficial effects of fluoride, as opposed to other developed countries where the importance of fluoride is well understood and prevention is more popular. 76% of teachers believe that the health of the mouth impacts the health of the body and 92% of teachers think that dental treatment is important as any other treatment which is similar to the 84.8% and 92.4% evaluated by Manjunath and Kumar [5].

Regarding prevention of dental problems, 92% of teachers feel that brushing prevents dental disease which is almost the same as previous studies done by Lang and Wolfolk [6] and Elena and Petr [7]. When asked which approach is the best for preventing tooth decay, 40% of instructors said that visiting the dentist every six months prevents dental decay, followed by brushing (36%), which is significantly less than earlier studies done by Lin et al. [10]. In comparison to the other research described above, there is a lack of awareness about the importance of dental plaque among school teachers, as previously stated.

At 48% of the time, teachers visit the dentist when they are in pain or have a toothache, compared to 19% in another study by Zhu et al. [9]. The reason for this is that in developing nations, pain is traditionally the primary motivator for visiting a dentist or doctor, which you can see in school teachers. You can also see from the results that oral health knowledge among school teachers is low, which is reflected in their attitudes. The majority of people believe that regular dental visits are necessary for proper tooth care, with 72% agreeing with Manjunath and Kumar's finding [5] of 69.4%. The most prevalent reason for going to the dentist is toothache (50%), compared to 19% in another study by Zhu et al. [9] followed by a friend's advice (20%). The reason for this is the same as stated above: school teachers' oral health knowledge is lacking, and they tend to view toothache as a major reason for going to the dentist rather than being concerned about any preventive measures. In a survey conducted by Ehzille et al. [11], 48% of school teachers said there is no specific reason for not seeing the dentist, followed by high expense, fear, and lack of time. Dentists are concerned with treatment but not prevention, according to 46% of instructors, which is identical to 45.5% in another study by Manjunath and Kumar [5]. They believe that certain dentists have been educated to treat problems forcefully and believe that this is the best approach. When compared to prior studies done by Tangade et al. [12], 62% said they brush twice each day, which is lower than 77.9%, and 34% stated they brush once per day, which is higher than 22.1%. This could be due to a misunderstanding that brushing twice is detrimental to the gingiva and teeth, which could be the cause of gum bleeding. 82% brush their teeth with a toothbrush and paste, which is similar to another study done by Varenne et al. [13]. 42% of school teachers change their toothbrushes every three months, which is similar to another study by Tangade et al. [12], which found 40% percent, and 26.8% percent every two months, which is less than another study by Zhu et al. [9], which could be due to the income factor because many school teachers, who have low income and are from low socioeconomic strata, tend to change their toothbrush every two months. 44% clean their teeth for more than 2 minutes, followed by 40% for 2 minutes. On the basis of qualification level, three groups were constituted as undergraduates, graduates, and postgraduates. Among them, postgraduates were found at the highest level for having good knowledge about oral health with a percentage of 17% followed by graduates with 14.9% and undergraduates with 8.5%. However, graduates obtained the highest percentage regarding fair knowledge about oral health as compared to undergraduates and graduates. A significant difference was found regarding knowledge about oral health on the basis of qualification (*P* value <0.05) and overall, the graduates were ranked highest for having fair knowledge as compared to undergraduates and postgraduates whereas undergraduates were ranked lowest for having the highest percentage on poor knowledge about oral health.

While assessing the knowledge about oral health on a gender basis, findings revealed significant differences. Females reported fair knowledge more than males as per the scores obtained by them: 77.2% for females and 68.5% for males. However, as compared to females, males had a higher percentage of good knowledge, while females had a higher percentage of poor knowledge about oral health. As per the scores, it is evident that gender affects the knowledge level regarding oral health and females are better on the fair level of knowledge while males score higher on the good level of knowledge as compared to females.

On the basis of age regarding knowledge about oral health, results show a significant difference. Four age groups were framed for data collection ranging 21–30 years, 31–40 years, 41–50 years, and >50 years. The respondents of the age group of 31–40 years showed the highest percentage (82.4%) regarding having fair oral health knowledge and practices. On the other side, the 21–30-year age group respondents obtained the least scores on poor level of knowledge. Findings show that, as the age increases from 21 to 40 years, the knowledge and oral health practices also increase simultaneously. But with the decrement of age, the level of knowledge and oral health practices also decreases. Thus, age affects these areas significantly.

School type also affects the knowledge towards oral health. Findings revealed that teachers of government and private schools have varied knowledge regarding oral health. Private teachers reported a higher percentage on a good basis of knowledge while an equal percentage was found for both private and government school teachers on a fair knowledge basis. On the poor knowledge level, only government teachers reported 16% whereas no percentage of private teachers. The study shows a significant difference between private and government school teachers towards oral health knowledge and practices and private teachers were found better in the above respect. On the basis of oral health practices and qualifications, this study reveals a significant difference among students. Postgraduates showed the highest percentage on the fair knowledge level and oral health practices. Undergraduates showed the highest percentage of good oral health practices and knowledge level. Equally, they were reported highest on the poor level of oral health knowledge and practices. A significant difference was found among the students of different levels in association with oral health practices and knowledge level. This shows that qualification level significantly affects oral health knowledge and practices.

The findings of the study also revealed the fact that age and gender also affect oral health practices and knowledge level. A significant difference was found among different age group teachers in association with oral health practices. On a gender basis, males were found better than females in regard to oral health practices. Similarly, as the age grows up, oral health knowledge and practices increase simultaneously until the age of >50. This shows a significant effect of age and gender on oral health knowledge and practices.

The type of school also affects the level of oral health knowledge and practices significantly. The government school teachers had good oral health practices as compared to private school teachers. On the other hand, private school teachers had higher fair and poor oral health practices as compared to government school teachers. A significant difference was found between private and government teachers on the basis of practices and type of school. The findings of the present study also support this fact.

The majority of undergraduate, graduate, and postgraduate respondents had reported a fair oral health attitude. The percentage of poor oral health among undergraduate to postgraduate teachers was higher than the percentage of good oral health among the same qualified teachers. A significant difference was found between the attitude and qualification of the teachers. Overall undergraduate teachers showed the highest percentage of good oral health knowledge and practices whereas postgraduate teachers showed the highest percentage on the poor level of oral health knowledge and practices. Thus, this result shows a significant effect of qualification and attitude of teachers towards oral health knowledge and practices.

School teachers were also assessed on their attitude and gender in regard to oral health knowledge and practices. The majority of males as compared to females showed a fair oral health attitude. Females had higher good attitude scores compared to males. This shows the significant effect of gender on oral health knowledge and practices.

Overall, the findings reveal the fact that age and gender affect the knowledge and attitude regarding oral health knowledge and practices significantly. Males are better in oral health practices whereas females have a positive oral health attitude. Similarly, as the age increases from 21 to 40 years, oral health knowledge and practices also increase but as the age reached up to 50 years, oral health knowledge and practices decrease as reported by the respondents. Qualifications of the teachers also affect their oral health knowledge and practices significantly but the difference between private and school teachers' oral health was found not significant.

Teachers have the potential to contribute positively to the process of developing and maintaining an effective school oral health program. School teachers as dental health educators have many advantages over the dental professionals [14]. They are able to instruct all school attending children rather than only seeking dental care.

School teachers have a daily influence on children at a time when the students are mounting their value systems. The close relationship built in classrooms allows teachers to individualize information to suit each child. The teachers are more skilled in education psychology than dental professionals and they can help their students to seek dental help at an early stage when the cost is low but the effectiveness is more.

The only possible disadvantage may be the inadequate background of teachers for providing oral health education as mentioned by Loupe and Frazier [15] and Peterson and Esheng [16]. Our country has an acute shortage of trained dental manpower, high levels of unmet dental needs, and a scarcity of economic resources.

To address this issue, it is prudent to effectively integrate school instructors into the oral health care delivery system. Efforts to educate teachers about the current preventive dentistry and the potential for oral health promotion among school instructors should be encouraged. Videotapes, games, and instructional brochures are examples of educational tools and aids that should be developed, implemented, and assessed. Teachers should be supported and encouraged to attend educational workshops on a regular basis [1]. The purpose of this study is to analyze school teachers' oral health knowledge, attitudes, and behaviors, as well as their oral health condition.

## 5. Conclusion

Overall, school teachers' oral health knowledge, attitude, and behavior are poor, with several of them unaware of the relevance of dental plaque and the role of fluoride in reducing dental caries. Their attitude is very similar to the general public, with several of them wanting to go to the dentist when they are in pain and unaware of any other oral cavity problems outside toothache. There is a disparity in knowledge and oral health practices among the study population, as several of them reacted as having good oral health practices, but we cannot wrap up the actual picture because we have not checked oral cavity parameters.

There is an essential need to increase oral health knowledge and attitudes among school instructors, including awareness of various oral cavity problems and how to avoid dental disorders. These improvements can be implemented by regular teacher training, such as oral health seminars, materials, and other similar ways. These basic steps assist and also exploit these teachers in transmitting oral health instruction to the youngsters, as we all know, they are heroes for the schoolchildren and the achievement of society as a whole.

## Figures and Tables

**Table 1:** Age and genderwise distribution of the study population.

			Gender		Total
			Male	Female	
Age	21–30 years	Count	53	88	141
		% within gender	24.9%	26.1%	25.6%
	31–40 years	Count	69	118	187
		% within gender	32.4%	35.0%	34.0%
	41–50 years	Count	66	90	156
		% within gender	31.0%	26.7%	28.4%
	>50 years	Count	25	41	66
		% within gender	11.7%	12.2%	12.0%

Total		Count	213	337	550
		% within gender	38.7%	61.3%	100.0%

**Table 2:** Genderwise distribution of study population according to the type of school.

		Type of school		Total
		Private	Government	
Male	Count	68	145	213
	% within type of school	24.7%	52.7%	38.7%

Female	Count	207	130	337
	% within type of school	75.3%	47.3%	61.3%

Total	Count	275	275	550
	% within type of school	100.0%	100.0%	100.0%

**Table 3:** Distribution of school teachers based on knowledge and type of school.

			Type of school		Total
			Private	Government	
Knowledge	Good	Count	57	27	84
		% within type of school	20.7%	9.8%	15.3%
	Fair	Count	202	204	406
		% within type of school	73.5%	74.2%	73.8%
	Poor	Count	16	44	60
		% within type of school	5.8%	16.0%	10.9%

Total		Count	275	275	550
		% within type of school	100.0%	100.0%	100.0%

*χ*2 = 23.791; *P*value <0.001 (significant).

**Table 4:** Distribution of school teachers based on knowledge and gender.

			Gender		Total
			Male	Female	
Knowledge	Good	Count	44	40	84
		% within gender	20.7%	11.9%	15.3%
	Fair	Count	146	260	406
		% within gender	68.5%	77.2%	73.8%
	Poor	Count	23	37	60
		% within gender	10.8%	11.0%	10.9%

*χ* ^2^ = 7.913; *P*value = 0.019 (significant).

**Table 5:** Distribution of school teachers based on their practices and type of school.

			Type of school		Total
			Private	Government	
Practices	Good	Count	11	53	64
		% within type of school	4.0%	19.3%	11.6%
	Fair	Count	215	184	399
		% within type of school	78.2%	66.9%	72.5%
	Poor	Count	49	38	87
		% within type of school	17.8%	13.8%	15.8%

Total		Count	275	275	550
		% within type of school	100.0%	100.0%	100.0%

*χ* ^2^ = 31.362; *P*value <0.001 (significant).

**Table 6:** Distribution of school teachers based on their attitude and type of school.

			Type of school		Total
			Private	Government	
Attitude	Good	Count	38	34	72
		% within type of school	13.8%	12.4%	13.1%
	Fair	Count	200	215	415
		% within type of school	72.7%	78.2%	75.5%
	Poor	Count	37	26	63
		% within type of school	13.5%	9.5%	11.5%

Total		Count	275	275	550
		% within type of school	100.0%	100.0%	100.0%

*χ* ^2^ = 2.685; *P* value = 0.261 (not significant).

**Table 7:** Comparison of oral health knowledge among government and private school teachers on the basis of right responses.

Questions	Government	Private
Knowledge about meaning of bleeding gums	143	154
How they protect themselves from bleeding gums	101	122
Knowledge about dental decay	110	153
Knowledge about dental plaque	75	99
Dental plaque leads to?	110	144
Knowledge about whether sweets affect oral health?	177	253
Health of mouth and teeth impacts the health of the body?	165	253
Knowledge about whether brushing teeth prevents dental diseases?	250	255
Knowledge about whether using fluoride strengthens the teeth?	143	152

## Data Availability

The data used are included within the article and may also be available by email to the corresponding author.

## References

[1] George B., John J., Saravanan S., Arumugham I. (2010). Oral health knowledge, attitude and practices of school teachers in Chennai. *Journal of Indian Association of Public Health Dentistry*.

[2] Singh P., Singh I., Gupta N., Tewari R., Agrawal D., Yadav P. (2015). Oral health knowledge, attitude, practices and oral health status among school teachers in and around lucknow UP. *IOSR Journal of Dental and Medical Science*.

[3] Prasai Dixit L., Shakya A., Shrestha M., Shrestha A. (2013). Dental caries prevalence, oral health knowledge and practice among indigenous Chepang school children of Nepal. *BMC Oral Health*.

[4] The World Oral Health Report (2006). *Continuous Improvement of Oral Health in the 21 Century-the Approach of the WHO Global Oral Health Programme*.

[5] Manjunath G., Kumar N. (2013). Oral health knowledge, attitude and practices among school teachers in kurnool-Andhra Pradesh. *Journal of Oral Health and Community Dentistry*.

[6] Lang P., Wolfolk M. W. (2001). Oral health knowledge and attitudes of elementary school teachers in Michigan. *Journal of Public Health Dentistry*.

[7] Elena B., Petr L. (2004). Oral health and children attitudes among mothers and school teachers in Belarus. *Stomatol Balt Dent Maxillofac Journal*.

[8] Al-Tamimi S., Petersen P. E. (1998). Oral health situation of schoolchildren, mothers and schoolteachers in Saudi Arabia. *International Dental Journal*.

[9] Zhu L., Petersen P. E., Wang H.-Y., Bian J.-Y., Zhang B.-X. (2005). Oral health knowledge, attitudes and behaviour of adults in China. *International Dental Journal*.

[10] Lin H. C., Wong M. C. M., Wang Z. J., Lo E. C. M. (2001). Oral health knowledge, attitudes, and practices of Chinese adults. *Journal of Dental Research*.

[11] Ehizele A., Chiwuzie J., Ofili A. (2011). Oral health knowledge, attitude and practices among Nigerian primary school teachers. *International Journal of Dental Hygiene*.

[12] Tangade P., Jain M., Mathur A., Prasad S. (2011). Knowledge, attitude and practice of dental caries and periodontal disease prevention among primary school teachers in Belgaum City, India. *Pesquisa Brasileira em Odontopediatria e Clínica Integrada*.

[13] Varenne B., Petersen P. E., Ouattara S. (2006). Oral health behaviour of children and adults in urban and rural areas of Burkina Faso, Africa. *International Dental Journal*.

[14] Flanders R. A. (2003). Oral health knowledge and attitudes of primary school teachers in different schools in Ankara. *Journal of The American Dental Association*.

[15] Loupe M. J., Frazier P. J. (1983). Knowledge and attitudes of schoolteachers toward oral health programs and preventive dentistry. *Journal of The American Dental Association*.

[16] Peterson P. E., Esheng Z. (1998). Dental caries and oral health behaviour situation of children, mothers and school teachers in Wuhan, People’s Republic of China. *International Dental Journal*.

